# Enhanced NMDA Receptor-Mediated Modulation of Excitatory Neurotransmission in the Dorsal Vagal Complex of Streptozotocin-Treated, Chronically Hyperglycemic Mice

**DOI:** 10.1371/journal.pone.0121022

**Published:** 2015-03-23

**Authors:** Eva C. Bach, Katalin Cs. Halmos, Bret N. Smith

**Affiliations:** Department of Physiology, University of Kentucky College of Medicine, Lexington, Kentucky, United States of America; University of São Paulo, BRAZIL

## Abstract

A variety of metabolic disorders, including complications experienced by diabetic patients, have been linked to altered neural activity in the dorsal vagal complex. This study tested the hypothesis that augmentation of N-Methyl-D-Aspartate (NMDA) receptor-mediated responses in the vagal complex contributes to increased glutamate release in the dorsal motor nucleus of the vagus nerve (DMV) in mice with streptozotocin-induced chronic hyperglycemia (i.e., hyperglycemic mice), a model of type 1 diabetes. Antagonism of NMDA receptors with AP-5 (100 μM) suppressed sEPSC frequency in vagal motor neurons recorded *in vitro*, confirming that constitutively active NMDA receptors regulate glutamate release in the DMV. There was a greater relative effect of NMDA receptor antagonism in hyperglycemic mice, suggesting that augmented NMDA effects occur in neurons presynaptic to the DMV. Effects of NMDA receptor blockade on mEPSC frequency were equivalent in control and diabetic mice, suggesting that differential effects on glutamate release were due to altered NMDA function in the soma-dendritic membrane of intact afferent neurons. Application of NMDA (300 μM) resulted in greater inward current and current density in NTS neurons recorded from hyperglycemic than control mice, particularly in glutamatergic NTS neurons identified by single-cell RT-PCR for VGLUT2. Overall expression of NR1 protein and message in the dorsal vagal complex were not different between the two groups. Enhanced postsynaptic NMDA responsiveness of glutamatergic NTS neurons is consistent with tonically-increased glutamate release in the DMV in mice with chronic hyperglycemia. Functional augmentation of NMDA-mediated responses may serve as a physiological counter-regulatory mechanism to control pathological disturbances of homeostatic autonomic function in type 1 diabetes.

## Introduction

The dorsal vagal complex of the caudal brainstem, including the nucleus of the solitary tract (NTS) and the dorsal motor nucleus of the vagus nerve (DMV), is a center for integrating neural and humoral signals regulating parasympathetic output to the viscera, including the digestive system. Primary viscerosensory afferents from peripheral organs and neural inputs from various brain regions synapse in the NTS. Neurons in the NTS integrate these various signals and transmit information to preganglionic parasympathetic motor neurons in the DMV, whose axons form the efferent limb of the vagus nerve [1–5]. By virtue of a network of fenestrated capillaries in the vagal complex, neurons in the NTS and DMV are also exposed to circulating molecules, including glucose, that can rapidly modulate neural activity [6–8]. Correspondingly, chronic hyperglycemia, as occurs in type 1 or type 2 diabetes, can alter vagal function and contribute to diabetes-associated visceral dysfunction [9–11].

Glutamate, the principal excitatory neurotransmitter in the vagal complex, activates ionotropic N-Methyl-D-Aspartate (NMDA) as well as both ionotropic and metabotropic non-NMDA receptors [12–15]. Upon activation by glutamate (in the presence of glycine), NMDA receptors typically contribute to membrane depolarization and Ca^2+^-dependent signaling cascades by increasing the conductance of Na^+^ and Ca^2+^ [16]. NMDA receptors are typically located on neuronal postsynaptic membranes, but they have also been identified on presynaptic terminals, where they modulate the release of GABA and glutamate [17–19]. NMDA receptors located on synaptic terminals (i.e., preNMDA receptors) are activated constitutively by ambient glutamate [17] and tonically-facilitate the release of glutamate, but not GABA, in the DMV [20]. Physiological consequences of activating NMDA receptors in the dorsal vagal complex in vivo include decreased hepatic gluconeogenesis, while their inhibition suppresses food intake [21, 22]. NMDA receptor function in the dorsal vagal complex is therefore critical for homeostatic regulation of vagal activity and visceral function.

The NTS contains a heterogeneous population of cells, comprised mainly of GABAergic and glutamatergic neurons, whose activity leads to synaptic inhibition and excitation, respectively, of DMV neurons and modulation of vagal motor function [23–25]. Increasing glucose concentration enhances glutamate release from viscerosensory vagal afferent terminals in the NTS [26] and inhibits DMV neurons[27]. Paradoxically, and separately from inhibitory effects, a sustained increase in glutamatergic EPSCs was observed in DMV neurons from hyperglycemic mice [28] in vitro, suggesting a chronic alteration in the synaptic regulation of vagal activity. The underlying mechanism(s) leading to this increased synaptic release of glutamate remain to be elucidated. NMDA receptors have been studied extensively for their role in modulating excitatory neurotransmission and synaptic plasticity under physiologic as well as pathologic states, in part by enhancing glutamate release from presynaptic terminals [16, 29, 30]. We hypothesized that changes in NMDA receptor function in central vagal circuitry contribute to persistently elevated glutamate release in the DMV and subsequent modulation of visceral function associated with systemic glucose dysregulation, a hallmark of diabetes.

## Methods

### Animals

All procedures were approved by the University of Kentucky Animal Care and Use Committee (Animal Welfare Assurance Number A3336–01) and were treated and cared for in accordance with National Institutes of Health guidelines. Euthanasia was accomplished by anesthesia with isoflurane to effect, followed by decapitation while anesthetized. Juvenile and young adult (24–65 days) female and male CD-1 (Harlan Laboratories, Indianapolis, IN) or GIN mice (FVB-Tg (GadGFP) 4570Swn/J; The Jackson Laboratory, Bar Harbor, ME) were used for all experiments and housed under a standard 14-h light-10-h dark cycle, with food and water provided without restriction. The GIN mice express EGFP in the somatostatinergic subset of GABA neurons in the NTS, which comprise a large proportion of NTS neurons [31]. The strategy of targeting recordings to non-EGFP labeled NTS cells in these mice was used to increase the probability of recording from VGLUT2+, glutamatergic neurons (NTS neuron phenotype was verified by RT-PCR, as described below).

### Animal Injections

**Fig 1:**
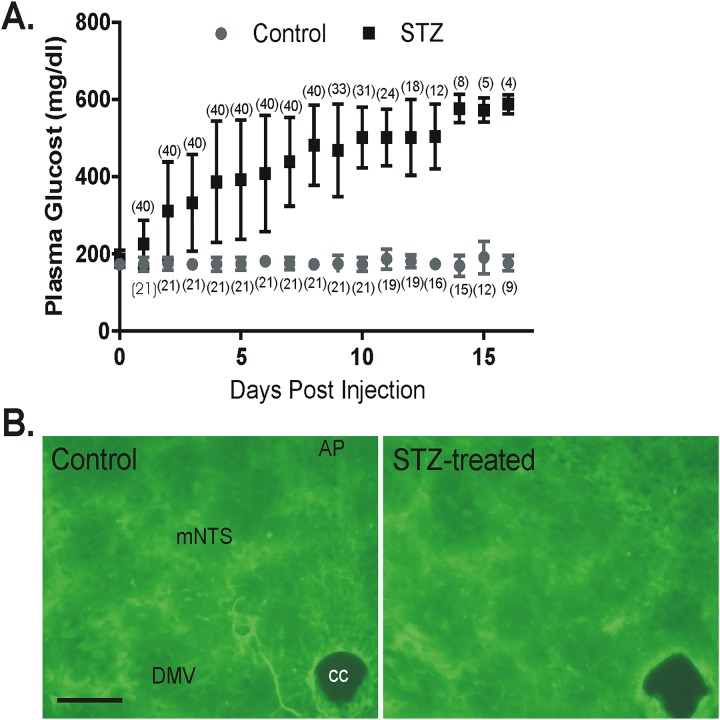
Effects of streptozotocin (STZ) injection on the time course of plasma glucose concentration and Fluoro-Jade B staining in the dorsal vagal complex. **A.** Time course of plasma glucose concentration (± standard deviation) in control and STZ-treated mice. Numbers in parenthesis indicate the number of mice maintained up to each day post injection. **B.** Fluoro-Jade B staining in the dorsal vagal complex 24 h after intraperitoneal injection of saline (left) or STZ (200 mg/kg; right). Cellular Fluoro-Jade labeling, an indicator of necrotic cell death, was not observed in the vagal complex after peripheral STZ injection. AP, area postrema; NTS, nucleus tractus solitarius; DMV, dorsal motor nucleus of the vagus; cc, central canal; scale bar = 100 μm.

Intra-peritoneal injections of streptozotocin (STZ; 200mg/kg in 0.9% NaCl; either a single dose or in 5 doses of 40 mg/kg each over 5 days), which kills insulin-secreting pancreatic β cells, was used to induce chronic hyperglycemia in mice. For single STZ injections, mice were fasted (4–6 h) prior to injection. Prior to STZ injections, blood glucose concentration (tail puncture) was measured using a Nova Max PLUS glucometer. Control mice were either injected with saline (0.9% NaCl) or untreated. No differences in electrophysiological parameters were observed between normoglycemic saline-injected and untreated mice; they were therefore pooled and considered as a single control group. Regression analysis of all parameters measured in the study showed no correlation between either drug effects or EPSC frequency with animal age (R^2^<0.25 for all measurements). Systemic glucose levels were measured daily. Onset of hyperglycemia (i.e., blood glucose level of above 300mg/dl) varied between animals, but occurred between 1 and 7 days post-STZ injection and remained elevated until the day of the experiment (Fig. 1A). Animals were used for electrophysiological recordings and molecular analyses after 7–10 days of continuous hyperglycemia. To determine potential neurotoxicity in the NTS of peripherally-injected STZ, a separate cohort of mice was injected with STZ as above (200 mg/kg; i.p.) and brainstem sections (30μm) were stained after 24 h using Fluoro-Jade B to label degenerating neurons, as described [32, 33]; no cellular labeling was detected (Fig. 1B). STZ-treated, hyperglycemic animals are referred to as hyperglycemic mice or identified as STZ-treated in graphical data depictions; normoglycemic animals are termed control mice.

### Brain stem slice preparation

Whole cell patch-clamp recordings were made using brainstem slices prepared from mice as described previously [20, 28, 31, 34]. Mice were anesthetized deeply by isoflurane inhalation to effect and then decapitated while anesthetized. The brain was removed and blocked on an ice-cold stand and the brainstem was glued to a stage for sectioning. Transverse (i.e. coronal) brainstem slices (300 μm) containing the dorsal vagal complex (from ~300 μm rostral to area postrema to the caudal edge of area postrema) were made in cold (0–2°C), oxygenated (95% O_2_–5% CO_2_) artificial cerebrospinal fluid (ACSF) using a vibrating microtome (Vibratome Series 1000; Technical Products, St. Louis, MO). These brainstem slices contain both the NTS and DMV, and preserve many intact synaptic connections between the two nuclei [25]. The ACSF contained (in mM): 124 NaCl, 3 KCl, 2 CaCl_2_, 1.3 MgCl_2_, 1.4 NaH_2_PO_4_, 26 NaHCO_3_, 11 glucose (pH 7.2–7.4; osmolality 290–315 mOsm/kg). For recordings, a single brain slice was transferred to a chamber mounted on a fixed stage under an upright microscope (BX51WI; Olympus, Melville, NY), where it was superfused continuously with warmed (30–33°C), oxygenated ACSF. Tetrodotoxin (TTX; 1–2 μM; Tocris Bioscience, Minneapolis, MN) was bath applied to record action potential-independent (i.e., miniature) excitatory postsynaptic currents (i.e., mEPSCs). Picrotoxin (100 μM; Sigma-Aldrich, St. Louis, MO) was added to the ACSF to block GABA_A_ receptors in all experiments. For specific experiments, DL-2-Amino-5-phosphonopentanoic acid (AP-5; 100 μM), NMDA (300 μM), and 6-Cyano-7-nitroquinoxaline-2,3-dion e (CNQX; 10 μM; all from Sigma-Aldrich) were added to ACSF. Since nicotinic receptors are abundant in the vagal complex and can interact directly with NMDA receptors [35–37], d-tubocurarine (DTC; 20 μM; Sigma) was also added in some experiments. Concentrations for receptor antagonists were derived from previous studies; 300 μM NMDA yielded maximal responses in preliminary studies in NTS neurons from control mice (data not shown). To avoid potential complications stemming from incomplete antagonist washout, AP-5 was applied to one cell per slice.

### Electrophysiological recording

Whole-cell voltage-clamp recordings were obtained in the DMV or NTS using recording pipettes pulled from borosilicate glass (open tip resistance of 3–5 MΩ; King Precision Glass Co., Claremont, CA). The pipette solution for most recordings contained (in mM): 130–140 Cs-gluconate, 10 HEPES, 1 NaCl, 1 CaCl_2_, 3 CsOH, 5 EGTA, and 2 Mg^2+^-ATP. Intracellular Cs^+^ was used as the primary cation carrier in voltage-clamp recordings to block K^+^ currents, including postsynaptic GABA_B_ receptor-mediated currents, in the recorded neuron. Neurons in the DMV and medial NTS were targeted for recording under a 40x water-immersion objective (numerical aperture = 0.8) with infrared-differential interference contrast (IR-DIC) optics, as described previously [28, 31, 34]. Electrophysiological signals were obtained using a Multiclamp 700B amplifier (Molecular Devices, Union City, CA), low-pass filtered at 2 or 3 kHz, digitized at 20kHz, and recorded onto a computer (Digidata 1440A, Molecular Devices) using pClamp 10.2 or 10.3 software (Molecular Devices). Seal resistance was typically 2–5 GΩ and series resistance, measured from brief voltage steps applied through the recording pipette (5 mV, 5 ms), was <25 MΩ (mean = 14.6±0.4 MΩ; n = 156) and was monitored periodically during the recording. Recordings were discarded if series resistance changed by >20% over the course of the experiment. Each recorded neuron represented an individual data point (n); recordings were made from at least four mice for each experimental group.

### RNA isolation

Two to three brainstem slices (300–600 μm) were isolated as described for electrophysiological recordings. The dorsal vagal complex, including most of both the DMV and NTS, was visualized under a dissecting microscope and excised from the rest of the brainstem with a razor blade. Resulting mini-slices were then suspended in 400–500 μL of TRIzol and gently shaken periodically for 5–25 min. Chloroform (100–250 μl) was added and tubes were vortexed for 15 s and then maintained at 4°C for 20 min and subsequently centrifuged at 12,000 rpm for 15 min at 4°C. The pellet was discarded and the RNA supernatant was transferred into fresh 1.5 ml centrifuge tubes, mixed with 500 μL of ice-cold propanol, incubated at room temperature for 10 min, and centrifuged at 12,000 rpm for 10 min at 4°C. Propanol was decanted and RNA was washed by re-suspension in 500 μL 75% ethanol followed by centrifugation at 7500–12,000 rpm for 10 min at 4°C. The wash step was repeated, the ethanol decanted, and RNA samples were air-dried for 10–20 min. RNA samples were re-suspended in 8–10 μL RNAse-free water and stored at -80°C or immediately reverse transcribed into cDNA. When obtaining single-cell mRNA from NTS neurons, the cytosol of the cell was aspirated into the recording pipette. The recording pipette carefully pulled away from the cell and its contents were expelled into a sterile centrifuge tube. RNA was stored at -80°C or immediately reverse transcribed into cDNA.

### TaqMan PCR

RNA samples were reverse transcribed in reverse-transcription master mix containing: 1 μl random nonamers (50 μM; Sigma-Aldrich), 5 μl MMLV RT buffer (5x) (Fisher Scientific, Pittsburgh, PA), 5 μl dNTPs (10 mM; Fisher), 2 μl DEPC-treated H_2_O (Fisher), 1 μl reverse transcriptase (Fisher), and RNAse inhibitor (1 μl; Fisher). For single-cell RT-PCR experiments, the cytosol of recorded NTS neurons was aspirated into the recording pipette at the conclusion of the electrophysiological recording and suspended in reverse-transcription master mix without enzymes and stored at -80°C until further use. Groups of cells were reverse transcribed after the addition of 1 μl reverse transcriptase (Fisher), and 1 μl RNAse inhibitor (Fisher) in a thermocycler (Mastercycler, Eppendorf) at 42°C for 90 min followed by 5 min at 95°C. For phenotype identification, positive controls (brainstem tissue samples) and single cells were probed for the presence of β-actin and vesicular glutamate transporter 2 (VGLUT2). Negative controls included ACSF or the contents of recording pipettes that contained extracellular constituents obtained after placing the recording pipet tip on the cell surface without breaking the membrane. No RNA was detected in these controls. Primers and probes for β-actin were: forward, CAGCAGGTACAGCATCACGG; reverse, GCCATGTACGTAGCCATCC; probe, CTGGTCGTACCACAGGCATTGTG; and for VGLUT2: forward, CCCGTCTACGCGATAATTGTT; reverse, GTCATGACAAGGTGAGGGACT; probe, ACTGCTCATCAGCCAGCTT. Master mix containing 1.5 μl MgCl_2_ (25 mM), 1.2 μl PCR buffer (10x), 0.5 μl dNTPs (10 mM), 0.5 μl each of forward primers, reverse primers, and probes (10 uM), 0.25 DNA polymerase, and 7.05 μl RNAse-free sterile H_2_O. 3 μl of single cell or positive control cDNA or RNAse free sterile H_2_O (for non-template control) was added to 12 μl master mix and loaded into optical tubes or a 96-well plate (Bio-Rad, Hercules, CA). Samples were centrifuged for 2 min at 1000 RPM and placed in an Applied Biosystems thermocylcer (ABI 7500; Life Technologies, Grand Island, NY) for PCR analysis. Samples were held at 95°C 2 min and cycled 50 times at 95°C for 30 s, 60°C for 15 s and at 72°C 15 s.

### NanoString

To quantify mRNA abundance in vagal complex mini-slices between control and hyperglycemic mice, the NanoString nCounter system was used. A custom designed codeset was designed by and purchased from NanoString Technologies (Seattle WA). This codeset included the seven known NMDA receptor subunits (NR1, NR2A-D and NR3A-B) as well as three housekeeping genes (β-actin, GAPDH, Hprt1). The isolated RNA quality assessment and concentration determination was made at the University of Kentucky Microarray Core Facility using the Agilent Bioanalyzer 2100 (Agilent Technologies, Santa Clara, CA). Average RNA integrity number (RIN) for samples used in this study was 9.4 ± 0.07 (Range: 8.9–9.7). 100 ng of total RNA was used from each sample. The hybridization reaction using the custom designed codeset, and nCounter master mix, was performed according to protocol by the Microarray Core Facility at the University of Kentucky using the nCounter (NanoString Technologies). The NanoString probe identifiers for genes analyzed were: For NR1, NM_008169.2:492; for NR2A, NM_008170.2:4080; for NR2B, NM_008171.3:6340; for NR2C, NM_010350.2:2720; for NR2D, NM_008172.2:1201; for NR3A, NM_001033351.1:1332; for NR3B, NM_130455.2:2030; for β-actin, NM_007393.3:1138; for GAPDH, NM_001001303.1:890; and for Hprt1, NM_013556.2:30. Normalization was performed using the nSolver software (NanoString Technologies). The nSolver software normalizes to negative and positive controls to eliminate background noise and variability unrelated to samples. All genes were subsequently normalized to the geometric mean of the three housekeeping genes to assess mRNA abundance for genes of interest.

### Western Blots

Brainstem slices (300–600 μm) were cut as described for electrophysiological recordings, the dorsal vagal complex was isolated from the slice, and mini-slices containing the DMV and NTS were immediately transferred to 40–60 μl of lysis buffer consisting of 0.15M NaCl, 5mM EDTA (pH 8), 1% Triton X-100, 10mM Tris-HCl (pH 7.4), 10μl/ml of 100mM PMSF (174.2 mg/10ml in methanol), and 100μl/ml of 0.5M NaF (pH 10). Each sample was sonicated and centrifuged immediately at 12,000 RPM for 3 min. Supernatant was aspirated, aliquoted, and stored at -80°C until further use. Protein concentration was measured using a Bradford Protein Assay. For Western blots, 20 μg of protein was loaded per lane. The appropriate volume of sample together with equal amounts of loading buffer was boiled in water for 2 min. Samples and ladder were loaded into precast SDS polyacrylamide gels and electrophoresed at 50 mA for 45–80 min. Proteins were then transferred at 200 mA for 2 hours onto polyvinylidene difluoride membranes for Western blot analysis. Membranes were blocked in 1:1 Odyssey blocking buffer/TBS/0.1% Tween 20 for 1 hr at room temperature. Due to well-separated molecular weights of the NR1 (band at 105–120 kD) and β-actin (band at 40–45 kD) protein membranes could be cut in half to be incubated over-night at 4°C, with a rabbit monoclonal anti-NMDAR1 (1:1000; Abcam, Cambridge, MA) and a rabbit monoclonal anti-β-actin (1:10000; Abcam) antibody in Odyssey blocking buffer/TBS/0.1% Tween 20. Membranes were washed 4 times (5 min) with TBS on a shaker and treated for 1 hr with fluorescence-conjugated anti-rabbit IgG (IRDye 680RD; Li-Cor Biosciences, Lincoln, NE). Membranes were then washed (4 x 5 min) and scanned on a densitometer (Odyssey model 9120, Li-Cor Biosciences) to quantify band density. Background density was subtracted from the NMDAR1 band density and normalized to β-actin, which was used as a loading control.

### Data analysis

Spontaneous and miniature EPSCs (typically 2-min continuous recording per condition) were analyzed with MiniAnalysis (Synaptosoft, Decatur, GA) to measure peak amplitude, frequency, and decay time constant. To measure current responses at membrane potentials between -80 and +30 mV, voltage steps were applied at 10 mV increments. Steps were of 1 second duration to reach saturating current responses for each voltage measured. Incremental current steps were made at inter-step intervals of 400 ms. Current measurements were made from averages of four runs and were measured at each potential at the end of the voltage step. Current-voltage response relationships were extrapolated from these measurements and compared between control and hyperglycemic mice.

Effects of drug application on sEPSC and mEPSC parameters within a recording were assessed using the non-parametric, intra-assay Kolmogorov-Smirnov (K-S) test. Grouped results of single comparisons of drug effects (i.e., before and after a single drug treatment) were tested for normality with the Shapiro-Wilk normality test and analyzed using a paired, two-tailed Student’s *t*-test when data were normally distributed or the Wilcoxon signed rank test for paired samples when normally-distributed population responses could not be established. When comparing pooled effects of a single variable between two animal groups (comparison between controls and STZ-injected mice) a homoscedastic two-tailed Student’s t-test or, in the absence of normally-distributed populations, the Mann-Whitney U test was used. The nature of responses from Western blot and Nanostring analyses did not produce normally distributed responses and the non-parametric Mann-Whitney U test was used to test for statistical significance. Statistical significance for all measures was set at *p*<0.05. Statistical measurements were performed with Microsoft Excel (Microsoft, Redmond, WA) or Prism (GraphPad Software, La Jolla, CA). Numbers were expressed as means ± SE.

## Results

### Increased glutamate release in the DMV of hyperglycemic mice

**Fig 2:**
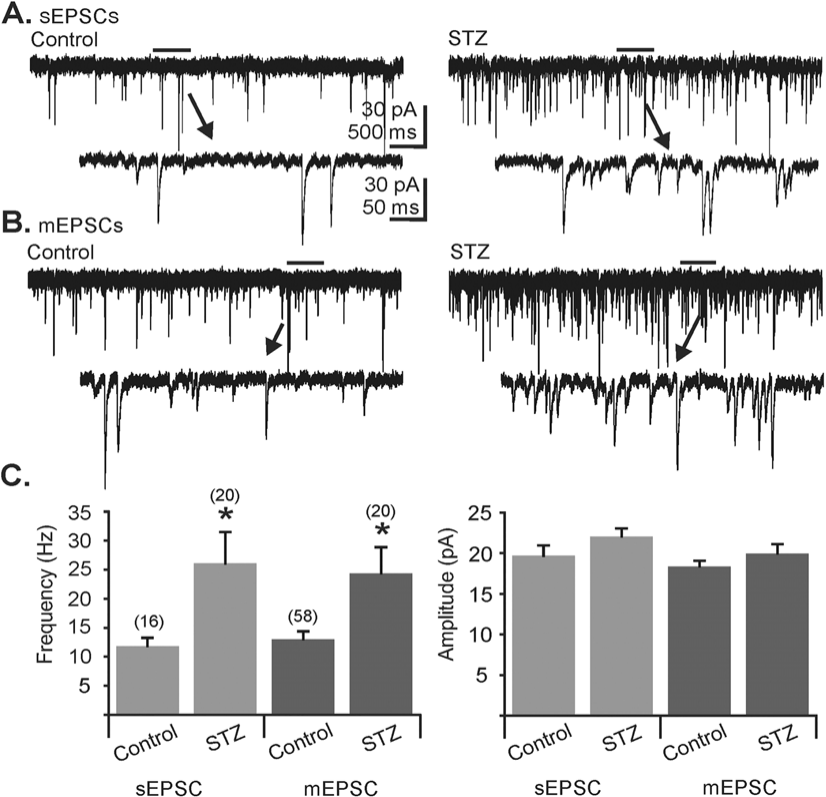
Comparison of sEPSCs and mEPSCs in dorsal motor nucleus of the vagus (DMV) neurons from control and STZ-treated, hyperglycemic mice. **A.** Representative traces showing sEPSCs in DMV cells from control (left) and hyperglycemic (STZ; right) mice. **B.** Representative traces of mEPSCs in neurons from control (left) and hyperglycemic (STZ; right) mice. Arrows point to expanded sections of traces indicated in **A** and **B**; picrotoxin (100 μM) was present in all recordings. **C.** Group frequency and amplitude graphs for sEPSCs and mEPSCs in control and hyperglycemic (STZ) mice (asterisk indicates significant difference from control; p<0.05; Mann-Whitney U test; numbers of replicates are indicated above each bar).

Whole-cell patch-clamp recordings were made from DMV neurons in slices from control and hyperglycemic mice, voltage-clamped at -80 mV in the presence of the GABA_A_ receptor blocker PTX (100 μM) to assess AMPA-mediated spontaneous and miniature EPSCs (i.e., sEPSCs and mEPSCs). Consistent with previous findings comparing relatively large populations of neurons [28], the frequency of sEPSCs was greater in hyperglycemic mice when compared to control animals (control: 11.80 ± 1.46 Hz, n = 16; hyperglycemic: 26.05 ± 5.44 Hz, n = 20; p<0.05; Fig. 2). In a separate cohort of neurons, tetrodotoxin (TTX; 1–2 μM) was added to the ACSF to record mEPSC frequency and amplitude. TTX blocks Na^+^ channels and the recorded mEPSCs are consequently action potential-independent events, representing the stochastic release of preterminal glutamate release. Similar to effects on sEPSCs, the frequency of mEPSCs (control: 13.33 ± 1.43 Hz, n = 58; hyperglycemic: 24.34 ± 4.65 Hz; n = 20) was also greater in hyperglycemic mice when compared to control animals (p<0.05; Fig. 2C). No difference in amplitude was observed for sEPSCs (control: 19.67 ± 1.29; hyperglycemic: 22.04 ± 1.01) or for mEPSCs (control: 18.36 ± 0.73 pA; hyperglycemic: 19.96 ± 1.16 pA; Fig. 2D). Consistent with previous reports [28], glutamate release was enhanced in the DMV of chronically hyperglycemic mice.

### NMDA receptor-mediated modulation of glutamate release in the DMV is enhanced in hperglycemic mice

**Fig 3:**
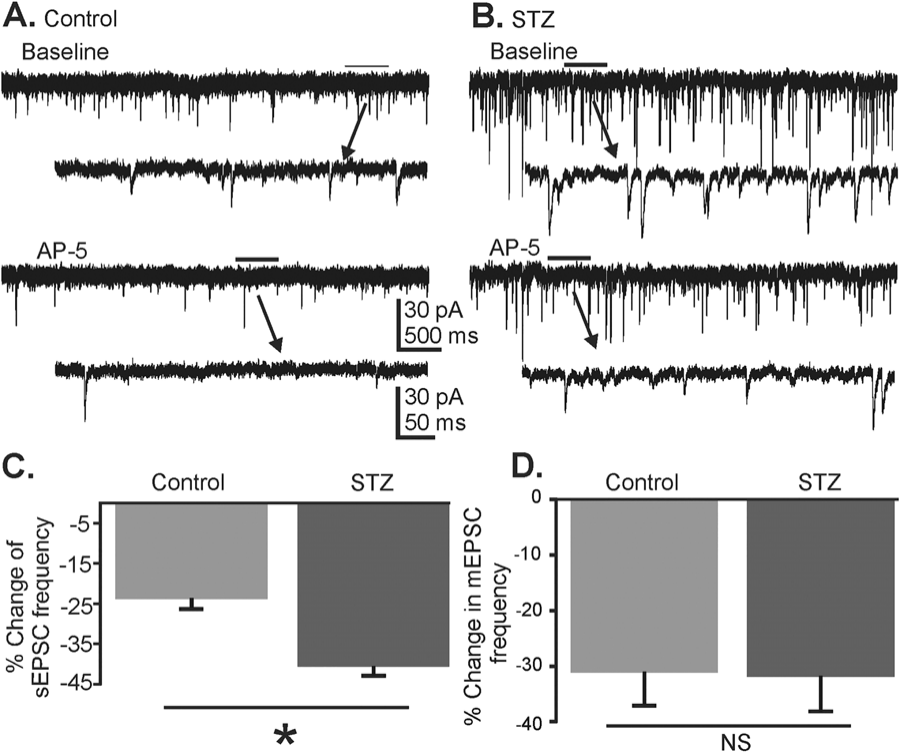
Effects of AP-5 application on EPSCs in control and hyperglycemic mice. **A.** Representative trace showing sEPSCs in a DMV neuron from a control mouse in control ACSF and during application of AP-5 (100 μM). **B.** Representative trace showing sEPSCs in a DMV neuron from a hyperglycemic mouse (STZ) before and during application of AP-5. Arrows point to expanded sections of traces indicated in **A** and **B**. **C.** Mean percent decrease in sEPSC frequency following application of AP-5 in control (n = 5) and hyperglycemic (n = 6) mice (asterisk indicates significant difference from control mice; p<0.05, Mann-Whitney U test). **D.** Mean percent decrease in mEPSC frequency following application of AP-5 in control (n = 9) and hyperglycemic (n = 10) mice. No difference was detected in the effect of AP-5 on mEPSC frequency in neurons from hyperglycemic and control mice (p = 0.97, Mann-Whitney U test).

The hypothesis that the function of tonically-active NMDA receptors is enhanced in the vagal complex of hyperglycemic mice, contributing to increased glutamate release in the DMV, was tested by assessing the effects of NMDA receptor blockade on AMPA receptor-mediated sEPSCs (i.e., no TTX) in recorded DMV neurons [20]. The response to application of the NMDA receptor antagonist AP-5 was compared between normoglycemic and hyperglycemic mice to reveal tonically-activated NMDA receptors. Randomly-selected DMV neurons were voltage clamped at -80 mV to diminish effects of postsynaptic NMDA receptor activation in the recorded DMV neuron and concentrate resultant effects on mechanisms mediated by receptors located on the somadendritic or synaptic terminal compartments of afferent neurons. Application of AP-5 in a subgroup of the cells presented in Fig. 2 resulted in a significant decrease in the frequency of sEPSCs in normoglycemic mice (Fig. 3A), reducing sEPSC frequency from a baseline of 13.42 ± 2.81 Hz to 10.08 ± 1.89 Hz during application of AP-5 (n = 5; p<0.05). Within-cell analysis using the K-S test showed a significant decrease in sEPSC frequency in each of the 5 cells (p<0.05). Application of AP-5 also diminished sEPSC frequency in DMV neurons from hyperglycemic, hyperglycemic mice (Fig. 3B) from a baseline of 16.85 ± 4.68 Hz to 10.42 ± 3.46 Hz during application of AP-5 (n = 6; p<0.05). A significant decrease was detected (K-S test) in each of 6 neurons from hyperglycemic mice (p<0.05). The relative change in sEPSC frequency in DMV neurons from hyperglycemic mice (40.65 ± 1.98% decrease; n = 6) was significantly greater than in cells from control mice (23.78 ± 1.74%; n = 5; p<0.05; Mann-Whitney U test; Fig. 3C). No change in sEPSC amplitude was observed in control (baseline 18.82 ± 1.93 pA and 18.83 ± 1.59 pA during application of AP-5; p>0.05) or hyperglycemic mice (baseline 20.46 ± 2.02 pA and 18.47 ± 2.11 pA during the application of AP-5; p>0.05). This result suggested that NMDA receptors exert a greater effect on glutamate release in the DMV of hyperglycemic than in normoglycemic mice.

### Preterminal NMDA receptors modulate glutamate release in both normoglyemic and hyperglycemic mice

Since sEPSCs reflect both action potential-dependent and -independent glutamate release, the NMDA receptor-mediated differences in sEPSC frequency observed between control and hyperglycemic mice could result from changes in receptors located on terminals contacting DMV neurons and/or soma-dendritic receptors on presynaptic neurons. Preterminal NMDA (i.e., preNMDA) receptors were previously identified in normoglycemic mice, which function to enhance glutamate release tonically in the DMV [20]. To assess preNMDA receptor involvement in tonic glutamate release, the effects of AP-5 (100 μM) were assessed in randomly-selected subgroups of the neurons from control and hyperglycemic mice. Neurons were recorded in the presence of TTX (1–2 μM) to block action potential-dependent synaptic activity and isolate effects on NMDA receptors located at synaptic terminals. Application of AP-5 decreased mEPSC frequency in DMV neurons of normoglycemic, control mice from a baseline of 12.84 ± 3.58 Hz to 8.97 ± 2.48 Hz in AP-5 (n = 10; p<0.05; Wilcoxon signed rank test; Fig. 3D). A significant decrease in mEPSC frequency in the presence of AP-5 was observed in each of 9 neurons from control mice (p<0.05; K-S test). No change in amplitude (19.75 ± 1.83 pA versus 19.23 ± 1.51 pA; p>0.05; Wilcoxon signed rank test) or decay time constant (1.74 ± 0.13 ms versus 1.68 ± 0.15 ms in AP-5; p>0.05; Wilcoxon signed rank test) was observed. In DMV neurons from hyperglycemic mice, application of AP-5 also resulted in a significant decrease in the frequency of mEPSCs (15.53 ± 4.96 Hz baseline and 9.70 ± 3.28 Hz during the application of AP-5; n = 10; p<0.05; Wilcoxon signed rank test). A significant decrease in response to AP-5 (p<0.05; K-S test) was observed in 9 of 10 neurons from hyperglycemic mice. Neither mEPSC amplitude (20.04 ± 1.74 pA versus 19.28 ± 1.43 pA in AP-5; p>0.05; Wilcoxon signed rank test) nor decay time (1.58 ± 0.12 ms versus 1.62 ± 0.09 ms in AP-5; p>0.05; Wilcoxon signed rank test) was altered. The relative effect of AP-5 on mEPSC frequency in neurons from control mice (31.12 ± 5.67% decrease; n = 9) was not different from that observed in neurons from hyperglycemic mice (31.90 ± 6.04% decrease; n = 10; p>0.05; Fig. 3D). These results indicated that preNMDA receptors modulate glutamate release in the DMV to a similar degree in both control and hyperglycemic mice. These results, therefore, were not consistent with altered function of preNMDA receptors in mediating the observed differences in NMDA receptor-mediated modulation of glutamate release in the DMV of hyperglycemic mice, but suggested altered NMDA receptor function on upstream glutamatergic neurons with intact projections to the DMV.

Although the numbers of cells in the subgroups used for intra-recording analysis of AP-5 treatment was too low to discern the statistical differences in EPSC frequency that were detected when the larger numbers of neurons were compared between groups (i.e., inter-recording analysis), baseline sEPSC and mEPSC frequency tended to be higher in the DMV of hyperglycemic mice, so the effect of AP-5 as a function of baseline mEPSC frequency was determined in the subsets of recordings in which effects of AP-5 were assessed. No correlation was detected between baseline frequency and relative AP-5-mediated response for sEPSCs (control mice, R^2^ = 0.12; hyperglycemic mice, R^2^ = 0.14) or mEPSCs (control mice, R^2^ = 0.02; hyperglycemic mice, R^2^ = 0.09). Thus, the relative effect of AP-5 was not a function of baseline EPSC frequency, consistent with altered NMDA receptor responses in hyperglycemic mice.

### Enhanced NMDA receptor-mediated whole-cell currents in NTS neurons from hyperglycemic mice

**Fig 4:**
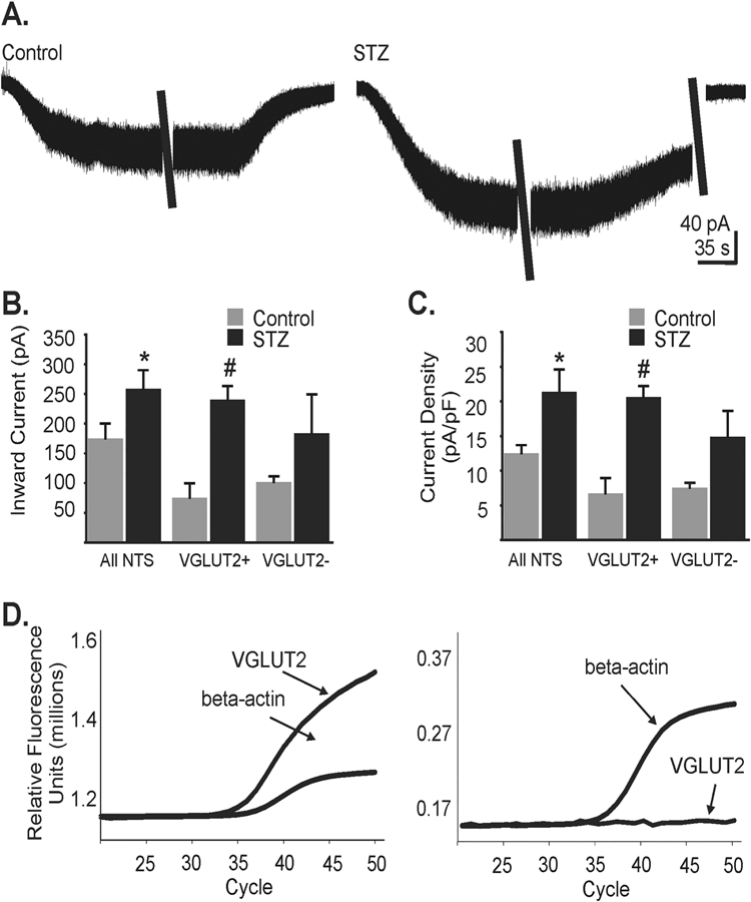
NMDA-mediated whole-cell current amplitude and current density in NTS neurons of control and hyperglycemic mice. **A.** Representative traces depicting whole cell current responses to application of NMDA (300 μM) in control and hyperglycemic mice (STZ). **B.** Average NMDA-mediated whole-cell current in control and hyperglycemic mice of the overall NTS neuron population sampled (n = 21 controls, n = 22 hyperglycemic), VGLUT2-positive neurons (VGLUT2+; n = 5 controls, n = 6 hyperglycemic) and VGLUT2-negative neurons (VGLUT2-; n = 4 controls, n = 5 hyperglycemic). * indicates significant difference from control (all cells; p<0.05; Mann-Whitney U test); # indicates significant difference from VGLUT2+ neurons in control mice (p<0.05; Mann-Whitney U test). **C.** Average NMDA-mediated current density in control and hyperglycemic conditions of VGLUT2+, and VGLUT2- NTS neuron populations. * indicates significant difference from NTS neurons from control mice (all cells; p<0.05); #, significant difference from control VGLUT2+ neurons (p<0.05). **D.** Examples of transcript detection using single-cell qRT-PCR for recorded VGLUT2+ and VLGUT2- neurons.

Since NMDA receptor responses in DMV neurons from control and hyperglycemic mice were different for sEPSCs, but not for mEPSCs, this suggested altered function of NMDA receptors located on the soma/dendritic region of intact, afferent glutamatergic neurons in hyperglycemic mice, but not of receptors located on glutamatergic terminals contacting DMV neurons. Glutamatergic NTS neurons form abundant intact, functional connections to DMV neurons in the coronal slice [25, 38], representing a candidate for effects of NMDA receptor-dependent modulation of glutamate release in the DMV. To determine if NMDA receptor-mediated function in upstream glutamatergic NTS neurons was increased in hyperglycemic mice, whole-cell currents were recorded from medial NTS neurons in response to applied NMDA (Fig. 4). Recordings were made from medial NTS neurons in GIN mice, targeting neurons that did not express EGFP (i.e., potentially non-GABAergic cells) in order to increase the likelihood of recording from glutamatergic neurons. To isolate NMDA receptor-mediated responses, all recordings were made with a Cs^+^-gluconate-based internal solution to block K^+^ currents and in the presence of TTX (1–2 μM), PTX (100 μM), strychnine (1–2 μM), DTC (20 μM), and CNQX (10 μM) to block action potentials and GABA_A_, nicotinic, and AMPA/kainate receptors, respectively. When applying 300 μM NMDA to NTS neurons recorded while voltage-clamped at a holding potential of -30 mV, significantly greater inward whole-cell current responses were observed in NTS neurons from hyperglycemic mice (259 ± 31 pA; n = 22) than controls (175 ± 25 pA; n = 21; p<0.05; Fig. 4A and B). To account for cell size, current density was calculated by normalizing individual responses to cell capacitance, which did not differ between animal groups (14.2 ± 0.8 pF controls, 13.2 ± 1.1 pF T1-diabetic; p = 0.45). When normalized to cell capacitance, NTS neurons from mice with chronic hyperglycemia had significantly greater NMDA-evoked current density (21.4 ± 3.2 pA/pF) than controls (12.5 ± 1.2 pA/pF; p<0.01; Fig. 4C).

Using TaqMan based single cell RT-PCR, a subset of NTS neurons recorded from both control and hyperglycemic groups was identified as glutamatergic (i.e., expressed VGLUT2; Fig. 4D). In this glutamatergic subpopulation of NTS cells, both NMDA-evoked whole-cell current (75.4 ± 24.3 pA controls; 258.0 ± 18.6 pA hyperglycemic; p<0.01) and current density (6.7 ± 2.2 pA/pF controls, 18.1 ± 2.4 pA/pF hyperglycemic mice; p<0.01) were significantly greater in neurons from hyperglycemic (n = 6) than normoglycemic control (n = 5) mice (Fig. 4B and C). Another subset of neurons expressed the housekeeping gene, β-actin, but expression of VGLUT2 was not detected (i.e., VGLUT2-negative; Fig. 4D). Current and current density responses to NMDA were not different between groups in VGLU2-negative neurons (p>0.05; n = 4 control and n = 5 hyperglycemic; Fig. 4B and C). This result suggests that NMDA receptor sensitivity was enhanced in glutamatergic NTS neurons from diabetic mice, consistent with the greater relative increase in glutamate release in the DMV of diabetic mice in response to AP-5 application.

### Voltage-dependence of soma-dendritic NMDA Receptors in NTS neurons

**Fig 5:**
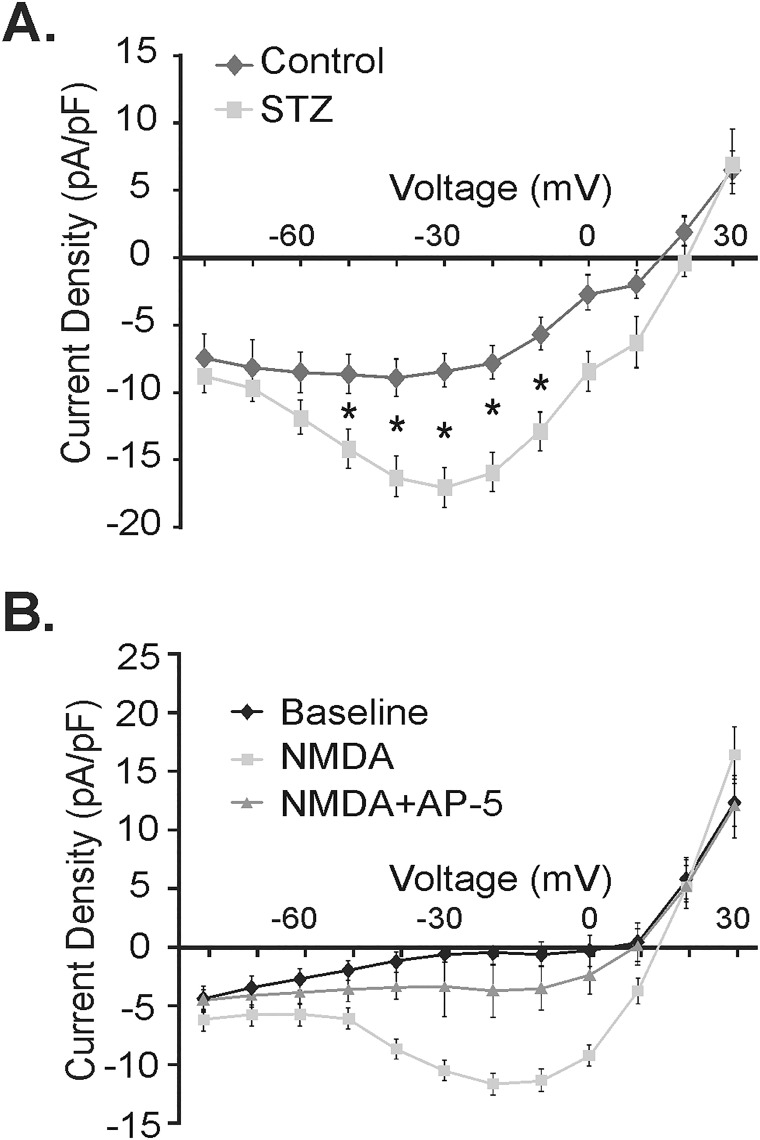
Current-voltage response relationships in NTS neurons from control and hyperglycemic mice. **A.** NMDA (300 μM) mediated current-voltage responses in control (n = 12 at -80 to 0 mV; n = 7 at -80 to 30 mV) and hyperglycemic mice (STZ; n = 21 at -80 to 0 mV; n = 9 at -80 to 30 mV). Asterisk indicates significant difference from control (p<0.05; Mann-Whitney U test). **B.** Current-voltage responses confirming AP-5 sensitivity of NMDA-mediated current-voltage responses (n = 3; 2 control and 1 hyperglycemic).

Responses to NMDA were determined in neurons voltage-clamped at -30 mV, a potential presumed to reveal maximal NMDA receptor-mediated responses [13, 39]. To determine if the voltage-dependence of the NMDA-induced responses differed between NTS neurons of control and hyperglycemic mice, a voltage step protocol was applied in a subset of neurons and currents evoked by NMDA application (300 μM) were examined at multiple voltages. NMDAR responses were recorded in the presence of PTX, TTX, strychnine, DTC and CNQX. The current-voltage relationship was determined during the maximal NMDA receptor-mediated response. When comparing the NMDA receptor-mediated current, calculated as the current density at each voltage in NMDA minus the current density at each respective baseline voltage, there was a significant increase in the current density in NTS neurons from hyperglycemic (n = 21) versus control (n = 12) mice at all voltages between -40 and 0 mV (p<0.05; Fig. 5A).

Maximal activation of the NMDA receptor is thought to occur near -30 mV due to Mg^2+^-dependent channel block [39], and our experimental results are therefore in agreement with this response being NMDA-mediated. The current-voltage (I-V) curves exhibited a nonlinear relationship, consistent with a voltage-sensitive response to NMDA (Fig. 5). The NMDA response was reversible (n = 8 controls; n = 11 hyperglycemic; not shown), and NMDA receptor specificity was confirmed using AP-5 in a subset of cells (2 control and 1 hyperglycemic), which blocked the response (n = 3; Fig. 5B).

### NMDA receptor subunit expression

**Fig 6:**
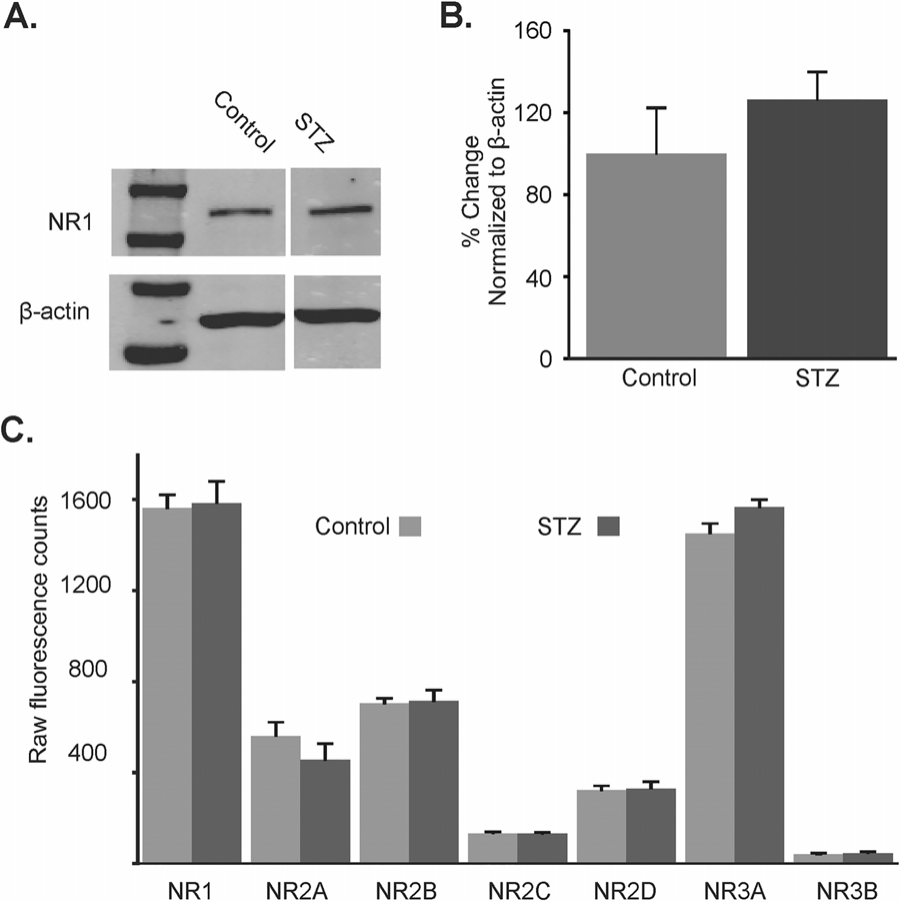
NMDA receptor subunit expression in control and hyperglycemic mice. **A.** Representative examples of NR1 subunit and β-actin protein expression in the dorsal vagal complex of control (n = 6) and hyperglycemic (STZ; n = 6) mice (bands to the left represent the protein ladder). **B.** Average NR1 protein expression in the dorsal vagal complex of control and hyperglycemic mice. **C.** NMDA receptor subunit mRNA abundance in control (n = 6) and hyperglycemic mice (n = 5), normalized to the three endogenous control genes β-actin, GAPDH and Hprt1.

The NR1 subunit is an obligatory NMDAR subunit [16]. An NR1 antibody raised against this subunit was used to examine protein levels (normalized to β-actin) as a measure of differences in the number of NMDA receptors in dorsal vagal complex mini-slices from control (n = 6) and hyperglycemic (n = 6) mice (Fig. 6A and B). There was no statistical difference detected between NR1 protein expression levels in normoglycemic control versus hyperglycemic mice (P>0.05).

The mRNA expression of the seven known NMDA receptor subunits was quantitatively assessed using nCounter NanoString technology. All genes were normalized to positive and negative internal controls. Genes of interest were subsequently normalized to the geometric mean of the three endogenous control genes, β-actin, GAPDH and Hprt1. No differences in mRNA expression were detected in any of the seven NMDA receptor subunits between control and hyperglycemic mice (Fig. 6C).

## Discussion

In this study, potential mechanisms underlying persistently enhanced glutamate release in the DMV of hyperglycemic mice were investigated. The EPSC frequencies reported here were observed in the presence of a GABA_A_ receptor antagonist to mitigate against possible GABA-mediated network interactions [13, 38, 40, 41] and potential hyperglycemia-induced GABA release [27] that might influence glutamate release in the DMV. Animals were maintained in a hyperglycemic state (i.e., ≥300 mg/dl blood glucose) for at least seven and up to 10 days before being used for electrophysiological and/or molecular experiments. Sustained hyperglycemia seems likely to play a role in inducing receptor plasticity, as it does in the hippocampus [42, 43]. Alternatively, insulin tends to inhibit glutamate release in the DMV[44], so the removal of insulin in this model by destruction of pancreatic β cells with STZ may also contribute to inducing changes in NMDA receptor function. It is also conceivable that STZ could alter neural function in the vagal complex through rapid neurotoxic effects of the drug, as occurs when it is injected intracerebroventricularly [32, 45, 46]. However, Fluoro-JadeB labeling did not reveal neuronal damage in the NTS in STZ-treated mice, implying that STZ-induced neurotoxicity did not contribute to functional NMDA response changes. Further, any direct, non-neurotoxic effect of STZ would have to be sustained (>10 days) if drug effects were responsible for the alteration in glutamate release and NMDA receptor function. Thus, altered NMDA receptor responsiveness in the mouse vagal complex seems likely to result from prolonged hyperglycemia/hypoinsulemia in this model of type 1 diabetes, although other possibilities exist due to potentially unappreciated effects of STZ.

The present results confirm the increase in mEPSC and sEPSC frequency in DMV neurons from hyperglycemic mice reported previously [28], consistent with the conclusion that glutamate release is enhanced in the DMV of mice after several days of chronic hyperglycemia, independent of potential changes in synaptic inhibition. Variability of EPSC frequency is inherently great between neurons in vitro, so detecting differences between groups required relatively large numbers of samples from each treatment group to identify statistically significant differences. The altered EPSC frequency, in the absence of a change in synaptic amplitude, is consistent with the hypothesis that excitatory neurotransmission is enhanced presynaptically, either at the level of the glutamatergic synaptic terminal or the soma-dendritic region of presynaptic glutamate cells contacting the DMV neuron. The increase in glutamate release persisted after the relative glycemic levels in slices between normo-and hyperglycemic mice were standardized to the levels in the ACSF used for recordings, suggesting that neural function in this region is fundamentally altered after several days of hyperglycemia/hypoinsulemia in STZ- treated mice.

Neurons in the vagal complex respond to acutely altered glucose concentration and their activity can also influence blood glucose content. Subsets of dorsal vagal complex neurons and primary viscerosensory synaptic terminals are glucose-sensitive [26, 27, 47, 48], consistent with longstanding evidence that glucose-sensing neurons in this region regulate both feeding and blood glucose concentrations [49, 50]. Acutely, injection of glucose into the vagal complex inhibits gastric motility and increases intragastric pressure, most likely by inhibiting activity of DMV neurons [27]. Acute changes in glucose concentration can also induce trafficking of serotonin 5-HT_3_ receptors on gastrointestinal primary vagal afferent neurons—but not those innervating the heart—to allow for rapid modification of primary gastric vagal afferent signals[51]. Many neurons recorded here were likely related to gastrointestinal function, but afferents relaying other types of visceral information also impinge on NTS neurons. Identifying the visceral association of neurons in which NMDA receptor responses are modified will be useful in identifying central effects of hyperglycemia with regard to specific parasympathetic functions. The glucose concentration used for the present experiments (11 mM) corresponds to ~198 mg/dl, which is within the normal blood glucose concentration range for fed control mice, but is lower than that for hyperglycemic mice. Notably, this is higher than the glucose concentration measured by microdialysis in other glucose-sensitive brain regions, like the ventromedial hypothalamus, in vivo (~1.4 mM) [52]. The dorsal vagal complex is inundated with fenestrated capillaries, and there is ample evidence to indicate that the blood brain barrier is permeable to large circulating molecules, including glucose, in this region [6–8]. This implies that glucose levels in the vagal complex may more closely reflect those found in the blood. Since the glucose concentration in the ACSF was identical in experiments from control and hyperglycemic mice (i.e., it was standardized), enhanced NMDA receptor-mediated responses probably reflect cellular changes that are maintained, even after glucose is normalized. Experiments to determine acute effects of glucose concentration on NMDA-mediated responses in hyperglycemic mice were not performed, but might yield information about temporal aspects of hyperglycemia-related NMDA receptor plasticity.

NMDA receptors have been studied extensively for their involvement in enhancing excitatory neurotransmission under pathological conditions [53, 54]. Activation of NMDA receptors located on glutamatergic synaptic terminals was identified previously as a means of modulating glutamate release tonically in the DMV [20]. Responses recorded in the presence of TTX indicated that preNMDA receptors were active tonically in both control and diabetic mice. The relative contribution of preNMDA receptors to tonic glutamate facilitation, however, was similar between cells from diabetic and control mice. This result argues against altered preNMDA receptor function contributing directly to the enhanced mEPSC frequency observed in hyperglycemic mice. When action potentials were not blocked, however, antagonism of NMDA receptors resulted in a greater decrease in sEPSC frequency in DMV neurons from hyperglycemic mice relative to normoglycemic controls, suggesting effects at soma-dendritic receptors of neurons with intact projections to the DMV.

The activity of DMV motor neurons is tightly regulated by synaptic inputs, largely arising from GABA and glutamate neurons of the adjacent NTS [23, 31, 38, 55], and functional, intact glutamatergic synaptic connections from NTS neurons to DMV motorneurons are maintained in the brainstem slice preparation used in this study [25, 55]. NMDA-mediated whole-cell current amplitude and current density was greater in NTS neurons of hyperglycemic mice relative to controls. The differences between groups were consistent in VGLUT2-expressing NTS neurons, indicating that increased NMDA receptor-mediated responses in glutamatergic NTS neurons represent a hallmark of altered dorsal vagal complex function in hyperglycemic mice. Further examination is required to determine if other endogenous properties of NTS glutamate neurons (e.g., membrane potential, input resistance, action potentials, etc.) are altered in mice with hyperglycemic.

Differences in response to NMDA between groups were also detected across the broader NTS neuronal population. The NTS includes a number of prominent phenotypes in addition to glutamate, including GABAergic, catecholeminergic, and peptidergic cell types. Using GIN mice, somatostatin-expressing GABAergic neurons could be identified in slices and were purposefully not targeted for recording in order to enhance the probability of recording from VGLUT2+ neurons. Consequently, the broader NTS population sampled represents a biased population in these studies, which omitted a large subset of inhibitory neurons. Whereas responses in this unidentified NTS neuron population exhibited significant differences between cells from normo- and hyperglycemic mice, the response amplitude in identified VGLUT2+ subset of neurons in hyperglycemic mice was less variable. In neurons identified as VGLUT2-negative (β-actin positive), a wide range of responses to NMDA application was observed and the overall NMDA effect was not different in VGLUT2-negative cells from hyperglycemic and control mice. Although NMDA response differences in other cell phenotypes cannot be disregarded, NMDA sensitivity in identified glutamatergic NTS neurons was significantly and consistently enhanced in mice with chronic hyperglycemia.

The possible mechanisms underlying the observed differences in NMDA receptor modulation of whole-cell current responses in NTS neurons from hyperglycemic mice are manifold. Metabolically-induced structural reorganization of glutamatergic synapses increases sEPSC frequency in hypothalamic neurons [56], and similar structural changes could contribute to the increased glutamate release observed in DMV neurons of hyperglycemic mice. The total number of NMDA receptors should be reflected by the expression of the NR1 subunit, since incorporation of this subunit is obligatory for functional NMDA receptor assembly [54]. But protein expression of the NR1 subunit was not statistically different between control and hyperglycemic mice, suggesting that large-scale increases in NMDA receptor-containing synapses did not occur. Likewise, receptor subunit reorganization on NTS neurons, which could affect channel conductance or Mg^2+^-sensitivity, should reflect altered mRNA levels for the respective subunits, which was also not detected. As a caveat to these findings, mini-slices of the dorsal vagal complex used for this analysis contained the entire dorsal vagal complex and parts of other dorsal brainstem regions. The lack of significant differences in NR1 protein or subunit mRNA expression between mini-slices from hyperglycemic and control mice may reflect, at least in part, the heterogeneous phenotype of the cell population included in mini-slices, especially if changes were restricted to the glutamatergic subpopulation.

Other factors, including posttranslational modification or trafficking of receptors could also be involved [16, 30]. NMDA receptor conductance is regulated by the activity of various kinases and phosphatase that alter receptor phosphorylation state, leading to a diverse array of functional changes in NMDA receptors, including prolonged open probability, increased conductance, and trafficking between membrane and intracellular compartments [57–59] and could likewise contribute to enhanced NMDA-mediated responses observed in NTS neurons from hyperglycemic animals. Receptor trafficking can occur relatively rapidly in the dorsal vagal complex [5, 28], and occurs in this model of type 1 diabetes, where TRPV1 receptors are internalized after several days of hyperglycemia/hypoinsulemia and can be trafficked to the membrane of synaptic terminals by exposure to insulin [28]. Enhanced responses to NMDA could likewise involve trafficking of NMDA receptors as a result of metabolic changes in hyperglycemic mice.

Vagally-mediated visceral function can be compromised in chronically hyperglycemic animal models or human patients with either type 1 or type 2 diabetes [9–11, 60]. Decreased parasympathetic visceral tone resulting from vagal atonia leads to a number of outcomes detrimental to maintenance of metabolic homeostasis, including elevated hepatic gluconeogenesis and diabetic gastroparesis (diabetic gastropathy). Normally, GABAergic input from the NTS to DMV neurons dominates the tonic regulation of vagal output, whereas glutamatergic regulation is thought to be more phasic in nature, being associated with specific viscerosensory stimuli [5]. Significant, sustained modulation of excitatory synaptic function in the DMV after several days of chronic hyperglycemia thus implies a fundamental change in the balance of synaptic input to vagal motor neurons, at least a portion of which is due to tonically-enhanced NMDA receptor function in glutamatergic NTS neurons that project to the DMV. NMDA receptor plasticity might therefore represent a homeostatic compensatory response to chronic hyperglycemia associated with type 1 diabetes. A sustained increase in glutamatergic, excitatory synaptic drive to DMV motor neurons in diabetic mice would be expected to enhance the tonic influence of glutamate in the DMV and increase vagal output, leading to diminished hepatic gluconeogenesis and/or enhanced output to the exocrine pancreas, as well as attenuation of diabetic gastroparesis [10, 11]. The persistent upregulation of glutamate release probably outlasts hyperglycemic periods and could contribute to continued visceral dysregulation after normalization of glucose levels in patients (e.g., by insulin). Chronic dysregulation of visceral autonomic control may contribute to development of insulin resistance and type 2 diabetes [61], underscoring the importance of understanding how to regulate vagal output when systemic glucose levels fluctuate. A better understanding of the mechanisms involved in potentiating NMDA receptor mediated function may provide clues to re-establishing autonomic control in diabetic patients.
